# Study of Allosteric Transitions of Human P-Glycoprotein by Using the Two-State Anisotropic Network Model

**DOI:** 10.3389/fmed.2022.815355

**Published:** 2022-02-09

**Authors:** Hongwu Li, Weikang Gong

**Affiliations:** ^1^School of Mathematics and Statistics, Nanyang Normal University, Nanyang, China; ^2^Faculty of Environmental and Life Sciences, Beijing University of Technology, Beijing, China

**Keywords:** two-state ANM, P-glycoprotein, allosteric pathway, multidrug resistance, ATP-binding

## Abstract

Human P-glycoprotein (P-gp) is a kind of ATP-binding cassette (ABC) transporters. Once human P-gp is overexpressed in tumor cells, which can lead to tumor multidrug resistance (MDR). However, the present experimental methods are difficult to obtain the large-scale conformational transition process of human P-gp. In this work, we explored the allosteric pathway of human P-gp from the inward-facing (IF) to the outward-facing (OF) state in the substrate transport process with the two-state anisotropic network model (tANM). These results suggest that the allosteric transitions proceed in a coupled way. The conformational changes of nucleotide-binding domains (NBDs) finally make the transmembrane domains (TMDs) to the OF state *via* the role of the allosteric propagation of the intracellular helices IH1 and IH2. Additionally, this allosteric pathway is advantageous in energy compared with other methods. This study reveals the conformational transition of P-gp, which contributes to an understanding of the allosteric mechanism of ABC exporters.

## Introduction

Long-term contact between cells and drugs can lead to multidrug resistance (MDR). It is also a major obstacle to cancer chemotherapy. MDR is mainly due to the overexpression of antineoplastic drug-efflux transporters (1). Such proteins belong to the ATP-binding cassette (ABC) superfamily and are widely located on the cell membranes. They utilize the energy of ATP hydrolysis to transport substrates across the lipid bilayer, even directly out of cells. The importance of studies on bacteria in elucidating several basic principles pertaining to ABC transporters is emphasized (2, 3). Interindividual differences in drug response are an important cause of treatment failures and adverse drug reactions. Human MDR protein 1, namely P-glycoprotein (P-gp), is an MDR ABC exporter that is widely distributed in the human body, it has a high-level expression generally in the blood–brain barrier and blood testosterone, liver, inner ear, and a variety of stem cells (4). Additionally, it shares a significant sequence identity with protein MsbA from gram-negative bacteria, which has been implicated in MDR. Researchers have found that P-gp has unusually broad polyspecificity, recognizing hundreds of hydrophobic substrates as 330–4,000 Da. It is like a “hydrophobic vacuum cleaner” pulling substrates from the membrane and expelling them to promote MDR. Therefore, it is of great importance to reveal the export process of human P-gp.

**Figure 1:**
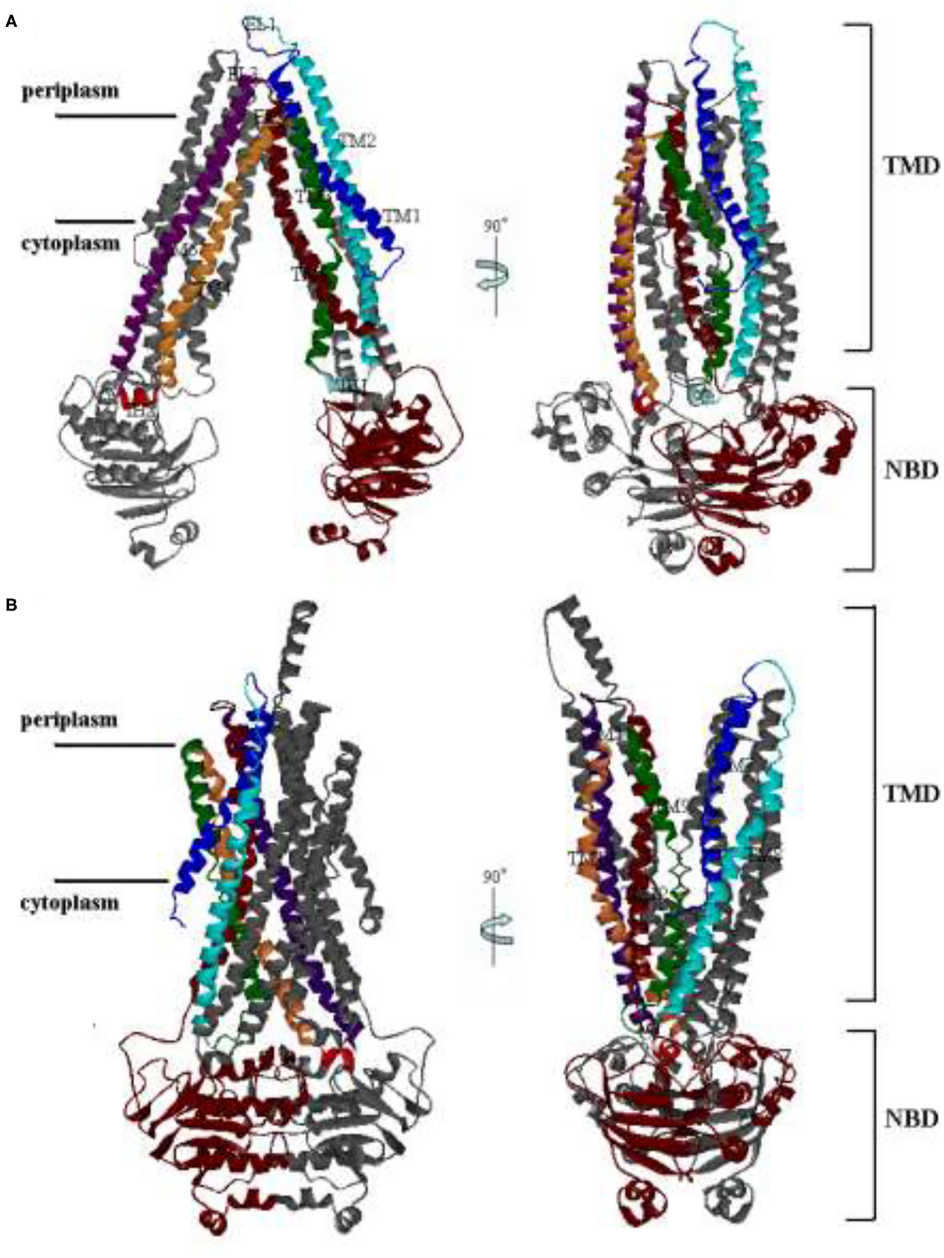
The structures of human P-glycoprotein (P-gp). **(A)** The conformation of the inward-facing (IF) state by the Swiss Model; **(B)** The conformation of the outward-facing (OF) state with O'Mara. The right panel is related to the left one by a 90° rotation around the axis shown.

P-glycoprotein has been studied as a hotspot for many years because of its clinical relevance. Especially, the structure of mouse P-gp, which has 87% sequence similarity to human P-gp, was obtained by Aller et al. Like mouse P-gp, human P-gp undergoes large-scale conformational changes between inward-facing (IF) and outward-facing (OF) state during a transport event (Figures 1A,B). It is a single polypeptide composed of 1,280 residues. It can be arranged as two pseudo homologous halves, named PA and PB (5). Each half (PA/PB) consists of a transmembrane domain (TMD) and a nucleotide-binding domain (NBD). Each TMD of human P-gp contains six helices, as labeled with TM1–TM6 for PA, TM7–12 for PB in Figure 1. Extracellular loops EL1, EL2, and EL3 of PA connect TM1–TM2, TM3–TM4, and TM5–TM6 in the periplasmic side, respectively. Intracellular helices IH1 and IH2 of PA connect TM2–TM3 and TM4–TM5 in the cytoplasmic side, respectively. The structure of PB is basically corresponding with PA. The TM helices cross each other to form a cavity in their interfaces. For the IF state, the TM helices are split into two groups forming two branches (TM1–3, 6, 10, 11 and TM4, 5, 7–9, 12) at the cytoplasmic side, resulting in the opening of the putative translocation pore to the cytoplasm (Figure 1A). Once the IF state is transferred into the OF state, the packing of TM helices will be rearranged. The helices TM3 and TM6 (TM9 and TM12) are crossed over to associate with the other branch. Thus, for the OF state, two branches are composed of helices TM1–2, 9–12, and TM3–6, 7–8, respectively. The TM1 is elongated to concur with an elongated helix in PA seen in the EM structure. TMDs interact with NBDs through IH1, 2 and IH3, 4. The NBDs of different transporters are highly conserved and responsible for ATP binding and hydrolysis. Each is composed of a RecA-like subdomain and a helical one. The RecA-like subdomain contains one conserved nucleotide-binding site; the helical subdomain includes the ABC family signature motifs. These subdomains constitute a head-to-tail dimer with nucleotide-binding sites at the interface. NBDs utilize the released energy of ATP hydrolysis to transport drug molecules from the inside to the outside of the cellular membrane, and this process accompanies a large-amplitude cooperation motion between the different structural domains of human P-gp.

A simple alternating access model was proposed by Jardetzky (6). In 2004, Higgins and Linton (7) conclusively proposed the ABC transporter conformation transformation model of “ATP-switch” based on a large number of experiments. At present, a lot of experimental information and conformational transformation models about human P-gp have been studied. In 1976, Juliano and Ling (8) first found that P-gp in the Chinese hamster ovary cells was selected for resistance to colchicines. In 1986, Chen et al. (9) discovered human P-gp for the first time. In 2008, Lee et al. (10) manufactured human P-gp of two-dimensional crystals in the phospholipid bilayer, then analyzed by using transmission electron microscopy (TEM) and got a low-resolution (14.6–74.5 Å) three-dimensional structure of human P-gp. Aller et al. (5) resolved three x-ray crystal structures of mouse P-gp with IF conformation in 2009. Later, Paul et al. (11) obtained the higher resolution crystal structures of mouse P-gp. Despite not getting a high-resolution crystal structure of human P-gp, the two have an 87% sequence identity (12). So, the crystal structures of mouse P-gp are chosen as good templates for human P-gp with IF conformation. In 2012, Wise (13) observed the coupled movement between NBDs and TMDs and referred it to the pronounced twisting of NBDs at the same time of closing. Chang et al. (14) explored the atomic detail of the conformational transmission of human P-gp by using targeted molecular dynamics (tMD) simulations and identified six key segments in the allosteric process. Recently, Pan and Aller (15) analyzed the conformational changes of ATP binding using all-atom molecular dynamics (MD) simulations. However, there is very little research on the large conformational transmission and analyses of the energy of human P-gp. Thus, the allosteric transition of human P-gp is still a hot issue.

All-atom MD simulation is an important tool to study protein dynamics and demonstrate their biological function. However, it is difficult to simulate the large-scale functional motion of transmembrane proteins and to obtain the complete information of conformational changes. To solve this problem, researchers put forward the coarse-grained model to steed up simulation with a freezing part of freedom degrees of the system. The Gaussian network model (GNM) (16) and the anisotropic network model (ANM) (17) are relatively simplified models based on the elastic network model (ENM) (18–21). Recently, Pan and Aller (15) analyzed the conformational changes and allosteric regulation of ATP binding by using all-atom MD simulations. Recently, Das et al. proposed a simple and computationally efficient method, the two-state ANM (tANM) (22) to construct a physically reasonable pathway between the two end points. First, a simple ENM representation is adopted for each of the end states, which accounts for the topology of inter-residue contacts in the structure. Second, a very simple two-state potential is constructed by mixing these two ENMs. The potential has a cusp hypersurface where the energies from both the ENMs are identical. Third, a minimum energy structure on the cusp hypersurface is searched and treated as the transition state. Fourth, starting from the transition state, two separate steepest descent minimizations were performed to connect the corresponding end states. Finally, the conformations collected from the two steepest descent paths along with the transition state provide a pathway. The Bahar group tested the Leucine transporter and Glutamate transporter, paving physically significant pathways, and helping generate experimentally testable hypotheses.

In this paper, the allosteric pathway between IF and OF state of human P-gp is explored through the tANM method; a reasonable allosteric pathway is obtained with the optimal energy. This helps to better understand the working mechanism of the human P-gp system.

## Materials and Methods

### Protein System

The x-ray structure of human P-gp in the IF state was modeled by using the Swiss-Model (http://swissmodel.expasy.org/) with a high resolution of mouse P-gp (PDB ID: 4Q9H) with the resolution value of 3.4 Å. The model of an OF conformation was obtained by O'Mara and Tieleman's group (23). The IF state contains residues from 34–630 and 701–1,275, opposite to 36–631 and 697–1,276 in the OF state, so we work it in mainly 1,170 residues of the two public sections.

### Adaptive ANM

For a protein with *N* residues, the configuration of the system is denoted by an elastic network, a node represents a residue, so ANM is an elastic network model defined around an experimental structure (e.g., x-ray or NMR structure) with the following energy function:


(1) $$\begin{array}{l} U_{ANM}(R)=\frac{\gamma }{2}\sum_{i<j}^{N}C_{ij}{\left(R_{ij}-R_{ij}^{0}\right)}^{2}=\frac{\gamma }{2}\sum_{i<j}^{N}C_{ij}{([\left(X_{i}-X_{j}\right)}^{2}\\ \;+{{\left(Y_{i}-Y_{j}\right)}^{2}+{\left(Z_{i}-Z_{j}\right)}^{2}]}^{1/2}-{R_{ij}^{0})}^{2} \end{array}$$

where γ is the uniform force constant, *N* is the number of residues, *R*_*ij*_ is the distance between the nodes *i* and *j*, $R_{ij}^{0}$ is the instantaneous distance between the nodes *i* and *j*, $R_{i}={\left[X_{i},Y_{i},Z_{i}\right]}^{}$ is the coordinate in *X, Y*, and *Z* directions of nodes *i*, *C*_*ij*_ is an element of the contact matrix defined by


(2) $$C_{ij}=\{\begin{array}{cc} 1 & if\;R_{ij}\leq r_{c}\\ 0 & otherwise \end{array}$$

*r*_*c*_ is the cutoff distance. *U*_*A*_(*R*) and *U*_*B*_(*R*) are, respectively, the ANM energy function in *A* and *B* states. The two-state potential function combines them with the following mixing rule (24):


(3) $$\begin{array}{r} U\left(R\right)=\frac{1}{2}\left(U_{A}\left(R\right)+U_{B}\left(R\right)-\sqrt{{\left[U_{A}\left(R\right)-U_{B}\left(R\right)\right]}^{2}}\right) \end{array}$$

### tANM Detailed Procedure

1. Two end structures are represented by the positions of their *C*_α_ atoms. These structures are aligned, and *M*-2 new intermediate conformers/images are generated by linearly interpolating between the end structures. The value of *M* is chosen by the user and is dependent on the value of the tolerance parameter ε^†^, which is the smallest energy difference between the two conformers that are considered to be different.
2. For each image, the energy is determined to use the two-state potential defined in formula (3). Then, the conformer *R*^†^ is identified for which energies from both the surfaces [i.e., $U_{A}\left(R^{†}\right)$ and $U_{B}\left(R^{†}\right)$] are equal within the tolerance parameter ε^†^.
3. Starting from *R*^†^, the transition state is searched by the following iterative procedure:
  (a) With appropriate choices of step-sizes *s*_*A*_ and *s*_*B*_ the knowledge of transition state for the present iteration *R*^†^(*n*), one step of the steepest descent minimization is carried out on each surface using the force of the respective surface and two new sets of coordinates *R*^*A*^(*n*+1) and *R*^*B*^(*n*+1) are generated, where
    (4) $$\begin{array}{r} R^{A}\left(n+1\right)=R^{\text{Ψ}}\left(n\right)+s_{A}f_{A}\left(n\right) \end{array}$$
    (5) $$\begin{array}{r} R^{B}\left(n+1\right)=R^{\text{Ψ}}\left(n\right)+s_{B}f_{B}\left(n\right) \end{array}$$
    and
    (6) $$\begin{array}{r} f_{A}\left(n\right)=-\frac{\partial U_{A}\left(R\right)}{\partial R}\;f_{B}\left(n\right)=-\frac{\partial U_{B}\left(R\right)}{\partial R} \end{array}$$
  (b) A linear interpolation (LI) is performed between *R*^*A*^(*n*+1) and *R*^*B*^(*n*+1) to find out the conformer that resides on the cusp hypersurface. This is a new approximation for the transition state, i.e., *R*^†^(*n*+1).
  (c) We iterate steps (3a) and (3b) until the energy difference between the two transition state conformers, obtained in two successive iterations, is less than tolerance ε_*conv*_.
4. Two separate steepest descent minimizations are performed, one on each surface, starting from the final transition state conformation $R_{f}^{†}$ and conformers, and conformers separated by using a user-defined RMSD are collected.
5. The cross-correlation between the displacements of the *i*th and *j*th residues at the *k*th conformer is defined as:
(7) $$\begin{array}{r} C_{A,ij}^{\left(k\right)}=\cos\left(h_{A,i}^{\left(k\right)},h_{A,j}^{\left(k\right)}\right) \end{array}$$
  where $h_{A,i}^{\left(k\right)}$ and $h_{A,j}^{\left(k\right)}$ are, respectively, the displacements of the *i*th and *j*th residues generated from the deformation vector:
(8) $$\begin{array}{r} h_{A,i}^{\left(k\right)}=\left(v_{A,3i-2}^{\left(k\right)},v_{A,3i-1}^{\left(k\right)},v_{A,3i}^{\left(k\right)}\right) \end{array}$$
(9) $$\begin{array}{r} h_{A,j}^{\left(k\right)}=\left(v_{A,3j-2}^{\left(k\right)},v_{A,3j-1}^{\left(k\right)},v_{A,3j}^{\left(k\right)}\right) \end{array}$$

The cross-correlation value ranges from −1 to 1. The positive values represent the residues moving in the same direction, and the negative values represent their movement in the opposite direction.

Several parameters listed above need to be specified before performing the calculation. The two-state potential function is characterized by the force constants and cutoff distances of ANMs. The ANM force constant does not affect the qualitative results (or the shape of conformational change driven by the normal modes) but uniformly scales the absolute size of motions. So, the force constants are inconsequential, and the cut-off distance is usually selected in the range of 12–16 Å. The values of ε^†^ are chosen to be in the range between 10e-4 and 10e-5. The most important parameters for an efficient implementation of the algorithm turned out to be the step sizes involved in the search of the transition state on the cusp hypersurface (and in step 3a). If step-sizes are too large, then the resultant movement of the transition state structure on the cusp hypersurface is large and the minimization algorithm does not work. On the other hand, if the chosen values are too small, then the convergence becomes slow.

In tANM, the cutoff is 13Å with the experimental information, the force constant is 0.1 kcal/(mol·Å^2^), the tolerance parameter ε^†^ is 5·10^−5^, and ε_*conv*_ is 10^−4^. The values of step sizes *s*_*A*_ and *s*_*B*_ are as quickly as possible to find the saddle point on the cusp hypersurface, so they set for 1 first, if the procedure spans the saddle point, turns down the value of step size, then calculates again from the breakpoint, until arriving the tolerance ε^†^, the RMSD of collecting conformers is 0.1 Å. The tANM has found an energy optimal path from opening to closing in physical.

## Results and Discussion

First, we aligned the two end structures by using VMD: (25), the original RMSD between them is 14.324 Å, and then used tANM to find the allosteric pathway of human P-gp.

### Sequence of Transition

**Figure 2:**
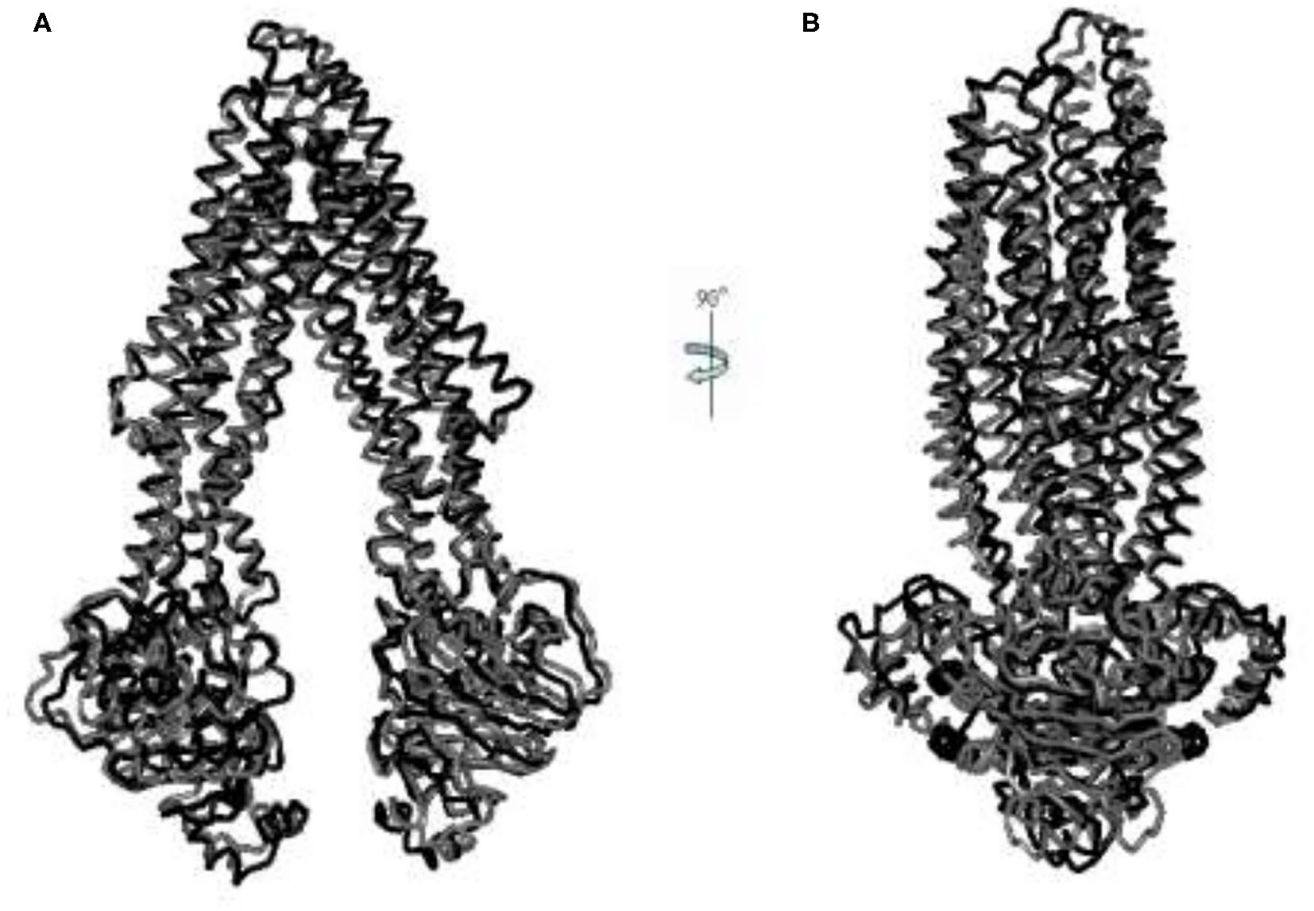
**(A,B)** The aligned figures of tr_39 and IF_2009. The RMSD of tr_39 (black) and IF_2009 (gray, template: 3G5U, resolution: 3.8 Å) is 4.026 Å.

After collecting conformers on the cusp hypersurface, we produced 171 intermediate conformers (tr_1-171), of which tr_124 is the conformer of the transition state. Also, we modeled the IF intermediate conformation (IF-II) by using mouse P-gp (PDB ID: 3G5U) with the resolution of 3.8 Å, corresponding to the structure of Wise (13) and Chang's et al. group (14). Further comparison of the conformers of an allosteric pathway with it, the lowest RMSD obtained between IF-II and tr_39 is 4.026 Å (Figure 2). To a certain extent, this illustrates the rationality of the pathway we got.

**Figure 3:**
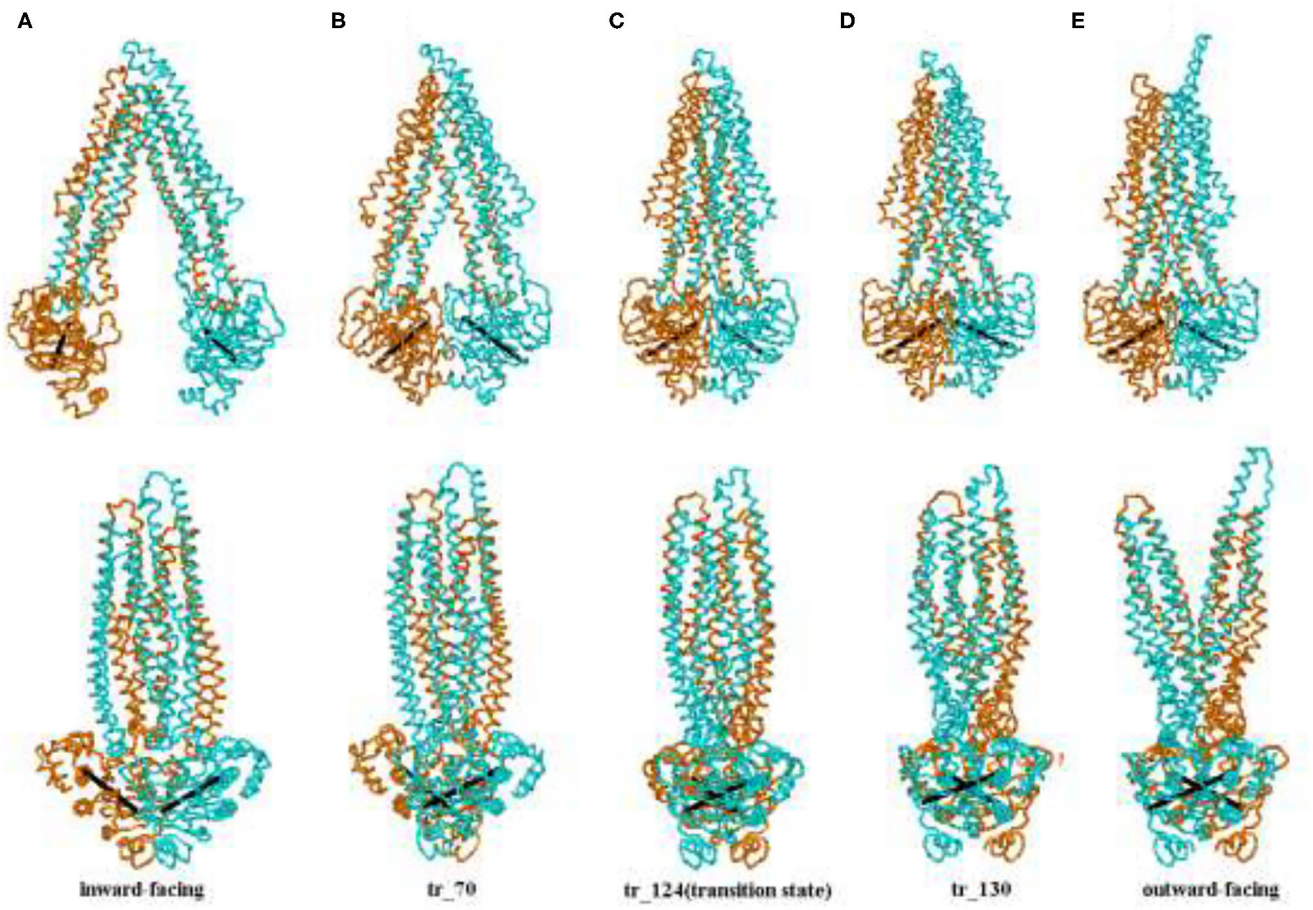
**(A–E)** Representative conformation sampling along the allosteric pathway. The lower snapshots are obtained by rotating the corresponding upper ones to 90° counterclockwise. The two lines, respectively, connect *C*_α_ atoms of the residues L413 and K536, E1059, and K1181 in two-nucleotide binding domain (NBD).

Figure 3 shows five representative snapshots along an allosteric pathway. Figure 3C (tr_124) is the transition state, that is to say, its energy is a saddle point on the cusp hypersurface. From Figures 3A–C (IF state), it can be seen that NBDs undergo an apparent conformational transition from the open to the nearly closed i ntermediates, and get affected due to this. The cytoplasmic side of the TMDs also experiences the corresponding changes. While for the periplasmic side, there are hardly evident conformational transitions. These results suggest that the allosteric transitions are more likely to be driven by NBDs, and this point is also hinted at in the following analyses about the changes of distances among some critical residues. Additionally, it is noticed that the relative orientation between the NBDs also changes and has a twisting motion although it is not very evident during this stage. In the transition state (Figure 3C), the cytoplasmic side of TMDs gathers into a cluster and NBDs become closed. From Figures 3C–E (the OF state), NBDs become completely closed. In addition, the periplasmic side of TMDs opens toward the outside. From Figure 3 (overall), we can see the whole transport process of human P-gp from IF to OF states. The sequence of transition of human P-gp is similar to exporter MsbA, which is in connection with their similar function and amino acid sequence (26). The sequence consistency of PA, PB part with MA, MB of MsbA are, respectively, 37, 34% by using the BLAST (12).

### Analysis of the Energy

**Figure 4:**
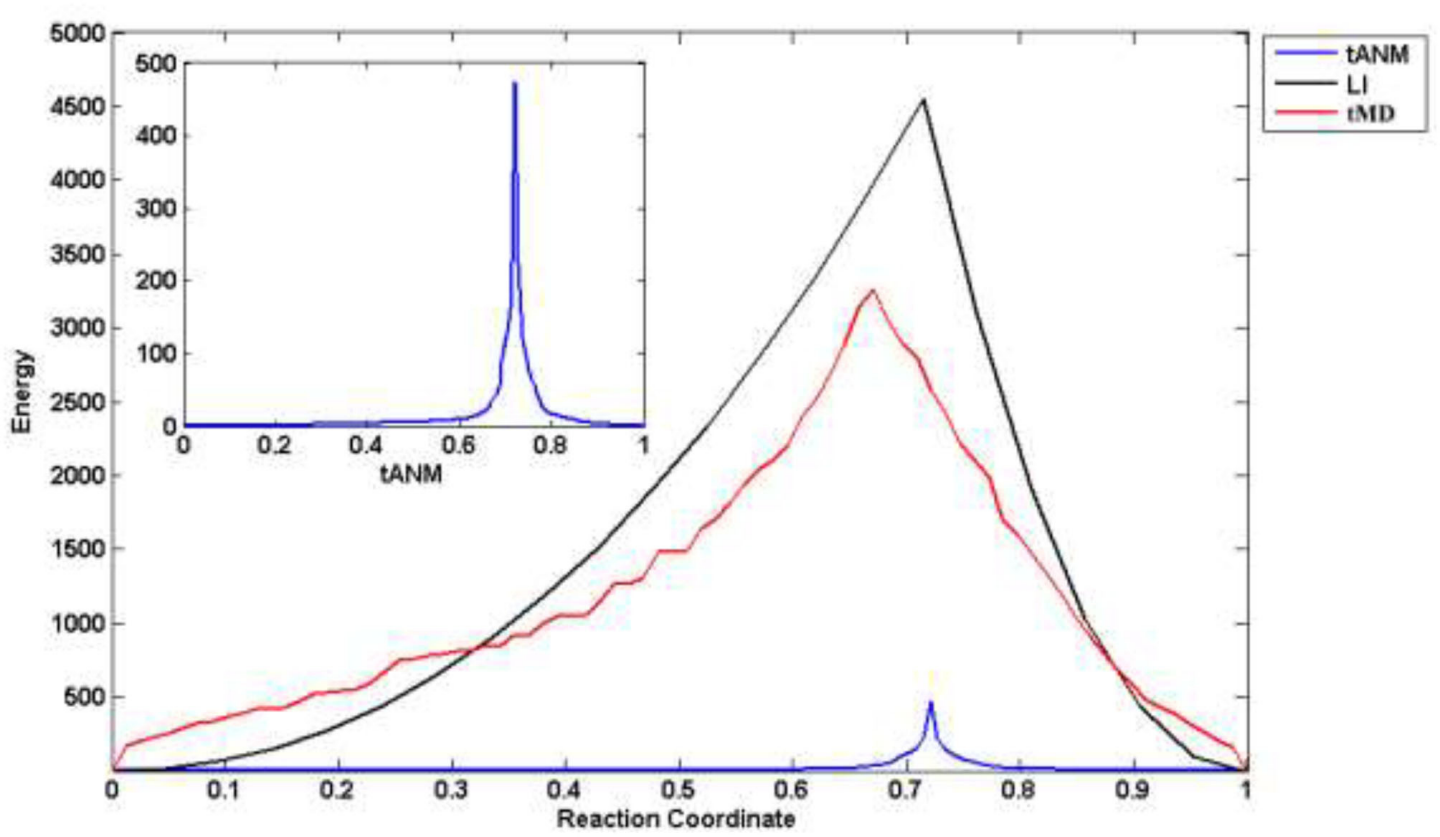
The allosteric energy comparison of different methods. X-axis is the reaction coordinate, that is, the projection of the cumulative displacement on the original distance vector.

We combined different simulation methods to analyze and compare changes in the allosteric energy of human P-gp. LI is a linear fitting with the coordinates of amino acids to get a user-defined number set of conformers between the two states, actually, this is a rigid allosteric process. tMD is based on traditional MD simulation to drive the coordinates of amino acids toward their target coordinates by means of extra potential. All TMD simulations were performed in the isothermal isobaric (substance, pressure and temperature, NPT) ensemble using the NAMD 2.8 software. The temperature was set to 310 K and kept constant during the simulation process using a Langevin thermostat with a damping coefficient of 1.0 ps^−1^. The two whole systems were first equilibrated for 0.5 ns and then conducted for 1 ns. In Figure 4, the reaction coordinate is the projection of the cumulative displacement $v^{\left(n\right)}=R^{\left(n\right)}-R_{A}^{\left(n\right)}$ on the original distance vector *d*^(0)^, that is, $x\left(n\right)=\frac{d^{\left(0\right)}\cdot v^{\left(n\right)}}{{\left|d^{\left(0\right)}\right|}^{2}}$, with IF and OF states representing the respective limits *x*(*n*) = 0 and 1. The energy peaks of the three methods are in the middle-late term of allosteric transmission. Compared with tMD and LI, tANM has the lowest energy consumption, the former two simulations are spanned a much taller energy barrier than tANM. To further observe tANM, it is slower in initial or final stages and faster before or after the transition state, which shows that low-frequency slow motions dominated the allosteric process of human P-gp in initial or final stages, then the participation of high-frequency local motions makes it rising rapidly.

### Changes of Distances Among Some Critical Residues During Transitions

**Figure 5:**
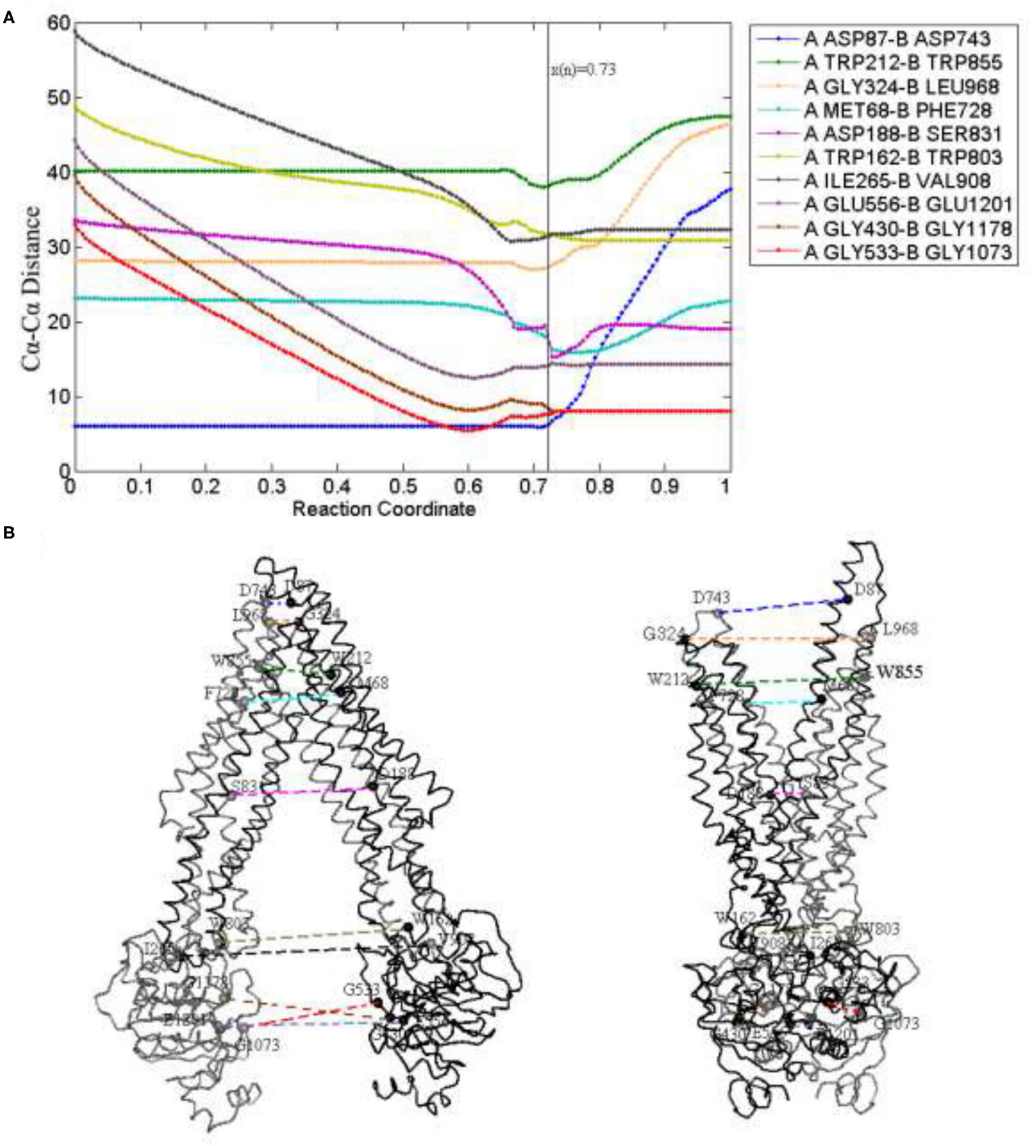
Distance changes **(A)** of some critical residue pairs **(B)** along the reaction coordinate. *x*(*n*) = 0.73 (black) is the reaction coordinate of the transition state. The color of lines in **(B)** is in correspondence to **(A)**.

To study the allosteric details of human P-gp, we analyzed the changes of distances among some critical residues during this transition (Figure 5).

For TMDs, the residue pairs PA ASP87-PB ASP743, PA TRP212-PB TRP855, and PA GLY324-PB LEU968 (located in the extracellular loops EL1,4, EL2,5, and EL3,6, respectively), are selected to indicate the movement of the periplasmic side. For the middle part of TMDs, the selected residue pairs are PA MET68-PB PHE728 and PA ASP188-PB SER831 located in the drug-binding sites. The residue pairs PA TRP162-PB TRP803 and PA ILE265-PB VAL908 (respectively, located in the intracellular helix IH1,3 and IH2,4) are used to reflect the changes in the cytoplasmic side (Figure 5B). For NBDs, mainly contain a number of critical conserved sequence motifs involved in ATP binding, namely walker A, walker B, and LSGGQ. So, the residue pairs PA GLY430-PB GlY1178, PA GLY533-PB GLY1073, and PA GLU556-PB GLU1201 are, respectively, selected to explain the distance changes of NBDs.

From Figure 5A, for the periplasmic side, the distances (PA ASP87-PB ASP743, PA TRP212-PB TRP855, and PA GLY324-PB LEU968) remain nearly invariant at first and gradually increase after the transition state, the periplasmic side is closed before the transition state, it comes to open in the late period of the whole transmission. Due to the elongation of a long helix structure TM1, the distance between the two EL1 leads to the biggest change in the later stage of a process. For the middle part, the residue pair MET68-PHE728 next to the periplasmic side interacts with the drug and the decrease of distance occurs mainly near the transition state, and as the TMDs open and drug release in the late process, it slowly increases. The distance of residue pair ASP188-SER831 decreases tardily at the beginning of the allosteric pathway and becomes obvious when the transmission nears the transition state, and because of TMDs open, it increases a little after the transition state and tends to be stable at the end of the pathway. The distances of residue pairs at the cytoplasmic side (PA TRP162-PB TRP803 and PA ILE265-PB VAL908) have an obvious decline trend before the transition state, it corresponds to the closing motion of NBDs and becomes stable in later stages.

Clearly, the closing speed of NBDs is more obvious than that of the TMD cytoplasmic part and much faster than that of the TMD middle part. These results imply that the allosteric transitions may be driven by NBDs. A previous study also indicates that the signals of conformational changes between the two NBDs are transmitted through the NBD–TMD interface to TMDs. When the two NBDs form dimers, an ATP-binding pocket (ABP) is composed of a Walker A and a Walker B with LSGGQ of another NBD. ATP molecules were sandwiched between the two NBDs and formed the “ATP sandwich dimers.” They are known as no. 1 and no. 2 ABP. The residue pairs PA GLY430-PB GlY1178 and PA GLY533-PB GLY1073 (respectively, located at no. 1 and no. 2 ABP) are closer to each other at the beginning of the allostery, and the distance decreases fastly, reaches consistently at the same time in the transition state, and remains stable until the OF state. The change of NBDs mainly occurs before the transition state, and the NBDs of the transition state have been closed, so the distance is basically invariant in the later stage. As to rise slowly of the distance before the transition state, we think it is also related to the twisting motion of NBDs. A specific discussion is given below.

### Change of Relative Position Between NBDs

**Figure 6:**
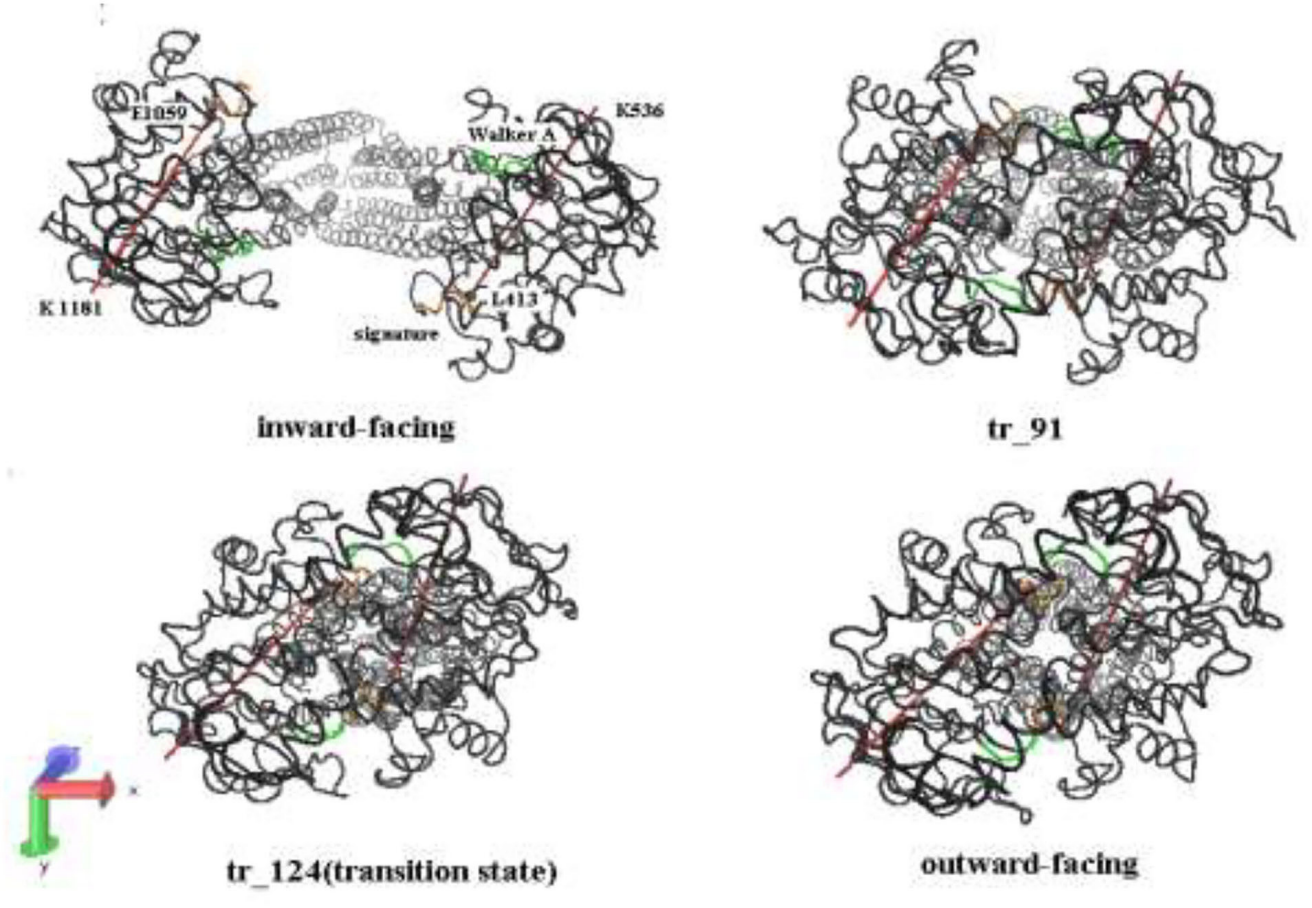
A bottom-up view of human P-gp in different transition intermediates. The red lines represent the distance of residual pairs PA L413-PA K536 and PB E1059-PB K1181 in NBDs. The green and orange, respectively, mark the Walker A and signature motifs in NBDs.

From the abovementioned analysis, in the allosteric process, the NBDs of human P-gp are not only close but also twisted (Figure 6). Here, we further analyzed a relative movement between the NBDs.

**Figure 7:**
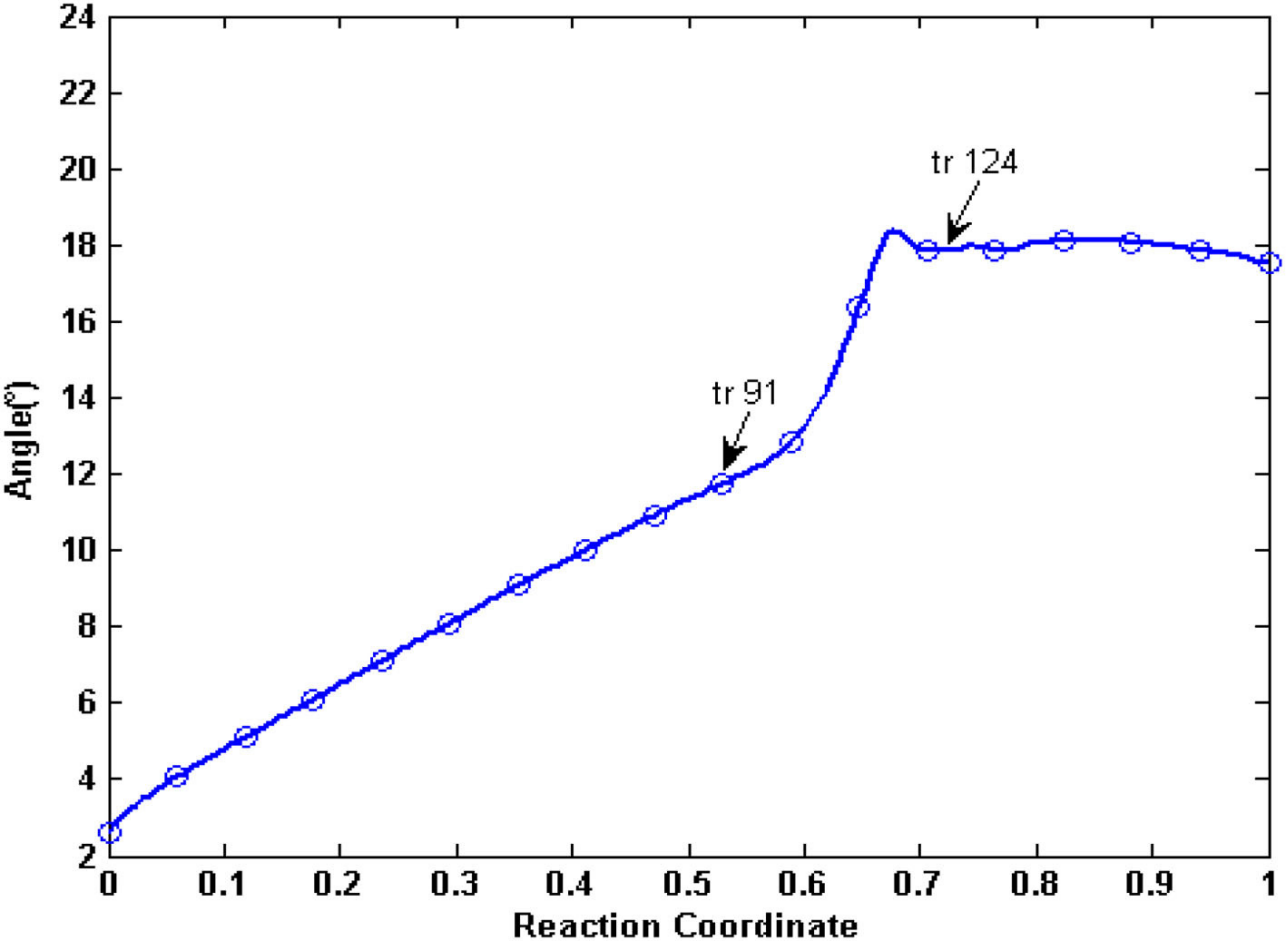
The angle changes of the two lines of PA L413-PA K536 and PB E1059-PB K1181. Arrows represent conformation 91 and conformation 124, respectively.

In the tANM simulation, each α helix and β -fold of NBDs basically remain unchanged, we selected the two lines to connect the residue pairs PA L413-PB K536 and PB E1059-PB K1181 and to illustrate the relative movement between the two NBDs (Figure 6). Figure 7 shows the allosteric degree change of acute angle that is crossed of two lines. We assumed if NBDs only do closing motion, the angle should be determined as invariable. From the IF state to the OF state of human P-gp, the intersection angle is closed to 0° and the two lines remain to be in parallel relationship with each other at the initial of allosteric transitions, due to the movement of NBDs, such as the relative position and angle change, therefore the twisting motions of NBDs have been involved at the initial of allosteric transitions. Then, the lines no longer remain basically parallel, the cross-relationship becomes obvious and the angle is also increasing. After the conformer of the 91st angle increases faster, meanwhile it is the most obvious moment of the twisting motion of NBDs in the whole allosteric transitions. The angle remains unchanged from the transition state to the OF state. In general, the relative movement between the NBDs results from a closing and twisting motion, and the twisting motion continues in the whole period of closing NBDs. From Figure 6, we also see that the relative movement makes NBDs form a closed dimer and two correct “ABP,” so the twisting motion is indispensable in the transformation of human P-gp.

### Coupling of the NBDs and TMDs

**Figure 8:**
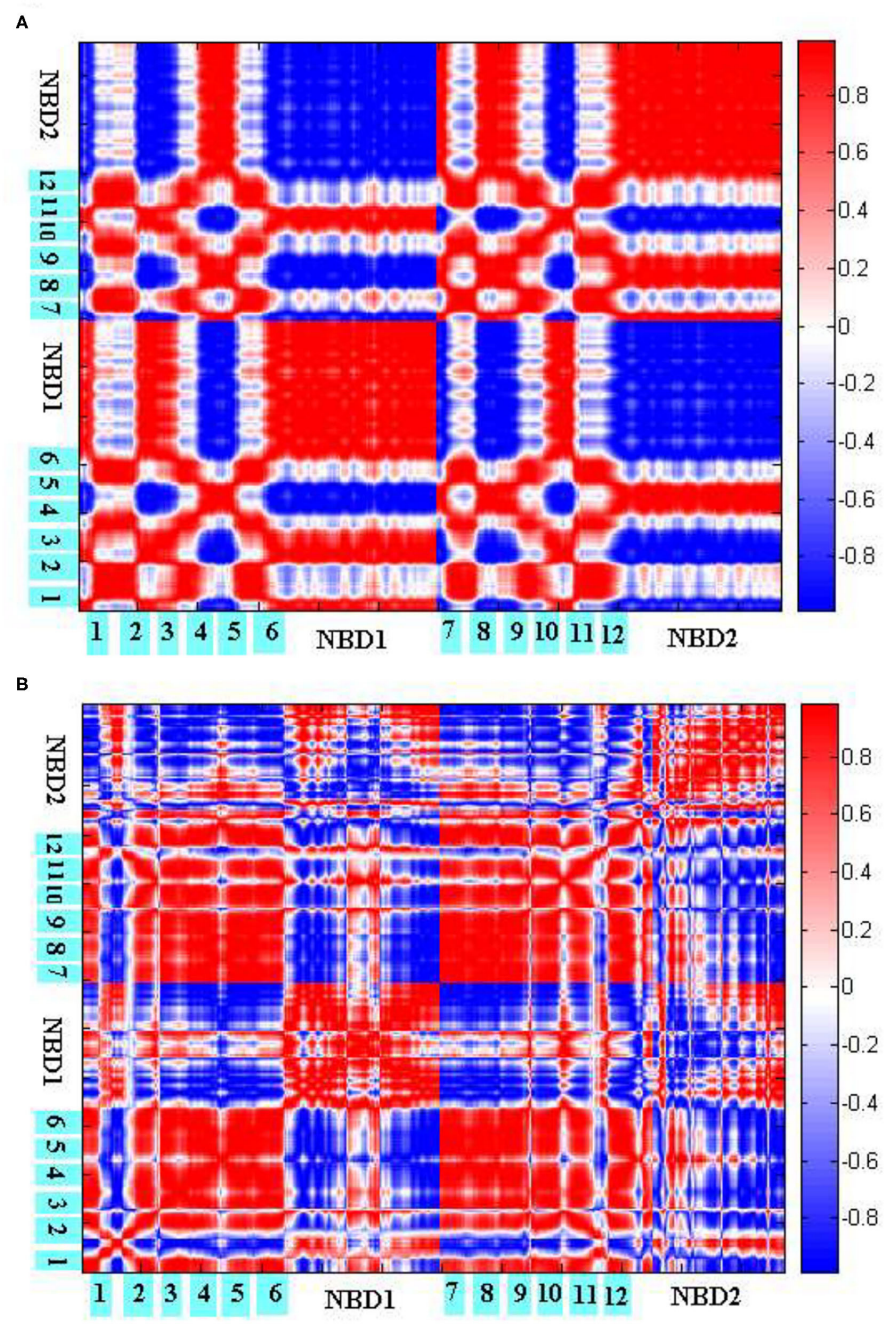
Cross-correlations between the residues of P-gp in the initial **(A)** and final **(B)** stages of allosteric transitions. The TM helices TM1-6 and NBDs are labeled out.

To explore the coupling mechanism between the different regions of human P-gp, we computed the cross-correlations between the residues of human P-gp according to Equations (7–9), respectively, in the initial (Figure 8A) and final (Figure 8B) stages of allosteric transitions. From Equations (7–9), the cross-correlations are computed from the recruited low-frequency eigenvectors obtained by ANM for the initial A and final B states. These eigenvectors reflect that the inherent motion modes are determined by the topological structure of the protein. Therefore, the following results of the cross-correlation analyses are closely related to the topological structures of human P-gp in different states.

Figure 8A reflects the coupling inside TMDs, between the NBDs, and between TMDs and NBDs in the initial stage. Overall, PA and PB move in a symmetrical way. For TMDs, they form two branches, with each branch consisting of helices TM1–3, TM6 from one half, and TM4–5 from the other. It is natural that the helices within one branch make strong positive correlations with each other. The correlations are mainly negative between the helices from different branches, which correspond to the closing movements of cytoplasmic and middle parts of TMDs. At the same time, because of the closing movements, both NBDs evidently make strong negative correlations with each other. For the coupling between TMDs and NBDs, it is observed that PA IH1 and IH2 make strong positive correlations with NBDs from PA and PB, respectively, which is consistent with the structural feature that both helices are deeply located into NBDs. In addition, the cytoplasmic parts of PA TM2, TM3 and TM4, TM5 influenced by IH1 and IH2, respectively, form intense positive correlations too with NBDs from PA and PB, respectively. However, their periplasmic parts, far away from IH1 and IH2, exhibit weak correlations with the corresponding NBDs. These results suggest that TMDs and NBDs move in a coupled way, and the helices IH1 and IH2 play an important mediating role. Additionally, TMDs may not move.

Figure 8B reflects the residual coupling of human P-gp in the final stage. Different from the initial stage, TMD helices are rearranged, with TM3 and TM6 splitting away from one branch to another, to adopt the OF conformation. In addition, because of an expanding outward movement, the periplasmic parts of TM1, TM2, and an extracellular loop EL1 make negative correlations with TMDs and positive correlations with NBDs. We could see that in Figure 8B TM1–2 makes a positive correlation with TM3–6 because they are consistent with their accumulations of the OF state. But, it is strange that the correlation of intramembrane PA TM3–6 with PB TM3–5 is positive, by comparing the conformers of the P-gp pathway, we can see these residues are connected closely to the intracellular helices IH1 and IH2, which may be related to a coupling effect. Due to the formation of the OF state, the intramembrane PB TM6 makes a negative correlation with another bifurcate half, the periplasmic parts of PB TM6 and NBD from PB exhibit a positive correlation with it. For NBDs, the correlation results show a positive correlation of the part of the RecA-like subdomain from PA with the part of the helical subdomain from PB because the conserved sequence motifs of them form the no. 1 ABP and are twisted together. The residues of no. 2 ABP are the same as those of no. 1. The other parts of NBDs still present a negative correlation, which indicates that the motion of NBDs happens in the whole conformational transition.

## Conclusions

Human P-gp transports intracellular drugs to the outside of cells, and the allosteric mechanism of human P-gp from IF to OF states remains unclear. In this work, the tANM was performed on the exporter human P-gp, we got a transition pathway with the advantage in energy and verified its rationality by using analysis. The results show that the allosteric transitions start from the large-scale closing motion of NBDs that is accompanied by a significant twisting movement between them. The twisting motion becomes more obvious in close to the transition state. The allosteric signal from NBDs is transmitted to TMDs through the intracellular helices IH1 and IH2. The twisting motion between the NBDs plays an important role in the packing rearrangement of TM helices, and the opening of the TMDs to the extracellular side which directly affects whether correctly form ABP. In addition, the movements of TMDs have non-rigid-body properties. This study revealed that the conformation transition of human P-gp could be helpful for human P-gp inhibitors and an understanding of the molecular mechanism of ABC exporters. The conformational rearrangement of human P-gp has been simulated from IF to OF states using targeted MD simulations in the future.

## Data Availability Statement

The original contributions presented in the study are included in the article/supplementary material, further inquiries can be directed to the corresponding author/s.

## Author Contributions

WG and HL designed the research. WG performed data analyses, wrote the manuscript, and developed the program. All authors revised and approved the manuscript.

## Funding

This work was supported by the Chinese National Science Foundation (31601379).

## Conflict of Interest

The authors declare that the research was conducted in the absence of any commercial or financial relationships that could be construed as a potential conflict of interest.

## Publisher's Note

All claims expressed in this article are solely those of the authors and do not necessarily represent those of their affiliated organizations, or those of the publisher, the editors and the reviewers. Any product that may be evaluated in this article, or claim that may be made by its manufacturer, is not guaranteed or endorsed by the publisher.

## References

[1] Goldstein LJ. Clinical reversal of drug resistance. Curr Prob Cancer. (1995) 19:65–124. 10.1016/S0147-0272(07)80004-3 7600845

[2] Higgins CF. ABC transporters-from microorganisms to man. Ann Rev Cell Biol. (1992) 8:67–113. 10.1146/annurev.cb.08.110192.000435 1282354

[3] Higgins CF. ABC transporters: physiology, structure and mechanism-an overview. Res Microbiol. (2001) 152:205–10. 10.1016/S0923-2508(01)01193-7 11421269

[4] Cascorbi I. Role of pharmacogenetics of ATP-binding cassette transporters in the pharmacokinetics of drugs. Pharmacol Therapeut. (2006) 112:457–73. 10.1016/j.pharmthera.2006.04.009 16766035

[5] Aller SG Yu J Ward A Weng Y Chittaboina S Zhuo R. Structure of P-glycoprotein reveals a molecular basis for poly-specific drug binding. Science. (2009) 323:1718–22. 10.1126/science.1168750 19325113 PMC2720052

[6] Jardetzky O. Simple allosteric model for membrane pumps. Nature. (1966) 211:969–70. 10.1038/211969a0 5968307

[7] Higgins CF Linton KJ. The ATP switch model for ABC transporters. Nat Struct Mol Biol. (2004) 11:918–26. 10.1038/nsmb836 15452563

[8] Juliano RL Ling V. Surface glycoprotein modulating drug permeability in chinese-hamster ovary cell mutants. Biochim Biophys Acta. (1976) 455:152–62. 10.1016/0005-2736(76)90160-7 990323

[9] Chen CJ Chin JE Ueda K Clark DP Pastan I Gottesman MM. Internal duplication and homology with bacterial transport proteins in the MDR1 (P-glycoprotein) gene from multidrug-resistant human-cells. Cell. (1986) 47:381–9. 10.1016/0092-8674(86)90595-7 2876781

[10] Lee J Urbatsch IL Senior AE Wilkens S. Nucleotide-induced structural changes in P-glycoprotein observed by electron microscopy. J Biol Chem. (2008) 283:5769–79. 10.1074/jbc.M707028200 18093977

[11] Ward AB Szewczyk P Grimard V Lee C Martinez L Doshi R. Structures of P-glycoprotein reveal its conformational flexibility and an epitope on the nucleotide-binding domain. Proc Natl Acad Sci USA. (2013) 110:13386–91. 10.1073/pnas.1309275110 23901103 PMC3746859

[12] Altschul SF Gish W Miller W Myers EW Lipman DJ. Basic local alignment search tool. J Mol Biol. (1990) 215:403–10. 10.1016/S0022-2836(05)80360-2 2231712

[13] Wise JG. Catalytic transitions in the human MDR1 P-glycoprotein drug binding sites. Biochemistry. (2012) 51:5125–41. 10.1021/bi300299z 22647192 PMC3383123

[14] Chang S Liu F Dong X Sun Y. Molecular insight into conformational transmission of human P-glycoprotein. J Chem Phys. (2013) 139:225102. 10.1063/1.4832740 24329094

[15] Pan L Aller SG. Equilibrated atomic models of outward-facing P-glycoprotein and effect of ATP binding on structural dynamics. Sci Rep. (2015) 5:7880. 10.1038/srep07880 25600711 PMC4389535

[16] Haliloglu T Bahar I Erman B. Gaussian dynamics of folded proteins. Phys Rev Lett. (1997) 79:3090–3. 10.1103/PhysRevLett.79.3090

[17] Atilgan AR Durell SR Jernigan RL Demirel MC Keskin O Bahar I. Anisotropy of fluctuation dynamics of proteins with an elastic network model. Biophys J. (2001) 80:505–15. 10.1016/S0006-3495(01)76033-X 11159421 PMC1301252

[18] Zhan M Li S Li F. Wavelet transformed Gaussian network model. J Theor Comput Chem. (2014) 13:14500539. 10.1142/S0219633614500539

[19] Ruvinsky AM Kirys T Tuzikov AV Vakser IA. Structure fluctuations and conformational changes in protein binding. J Bioinf Comput Biol. (2012) 10:12410028. 10.1142/S0219720012410028 22809338 PMC3401964

[20] Togashi Y Flechsig H. Coarse-grained protein dynamics studies using elastic network models. Int J Mol Sci. (2018) 19:3899. 10.3390/ijms19123899 30563146 PMC6320916

[21] Anand DV Meng Z Xia K. A complex multiscale virtual particle model based elastic network model (CMVP-ENM) for the normal mode analysis of biomolecular complexes. Phys Chem. (2019) 21:4359–66. 10.1039/C8CP07442A 30724932

[22] Das A Gur M Cheng MH Jo S Bahar I. Exploring the conformational transitions of biomolecular systems using a simple two-state anisotropic network model. PLoS Comput Biol. (2014) 10:e1003521. 10.1371/journal.pcbi.1003521 24699246 PMC3974643

[23] O'Mara ML Tieleman DP. P-glycoprotein models of the apo and ATP-bound states based on homology with Sav1866 and MalK. FEBS Lett. (2007) 581:4217–22. 10.1016/j.febslet.2007.07.069 17706648

[24] Maragakis P Karplus M. Large amplitude conformational change in proteins explored with a plastic network model: adenylate kinase. J Mil Biol. (2005) 352:807–22. 10.1016/j.jmb.2005.07.031 16139299

[25] Humphrey W Dalke A Schulten K. VMD: visual molecular dynamics. J Mol Graph. (1996) 14:33–8, 27–8. 10.1016/0263-7855(96)00018-5 8744570

[26] Seyffer F Tampe R. ABC transporters in adaptive immunity. BBA Gen Sub. (2015) 1850:449–60. 10.1016/j.bbagen.2014.05.022 24923865

